# Shock in the Setting of Diamond-Blackfan Anemia Relapse

**DOI:** 10.1155/2021/6623119

**Published:** 2021-04-06

**Authors:** Metri Haddaden, Samir Husami, Modar Alom, Yifan Pang, Zaid Imam, Daniel Keena

**Affiliations:** ^1^MedStar Health, Department of Internal Medicine, Baltimore, Maryland, USA; ^2^Department of Medicine, Division of Nephrology, Washington University School of Medicine, St. Louis, Missouri, USA; ^3^National Cancer Institute, National Institutes of Health, Bethesda, Maryland, USA; ^4^Department of Medicine, University of Toledo-College of Medicine and life Sciences Toledo, OH, USA; ^5^William Beaumont Hospital, Department of Internal Medicine, Royal Oak, Michigan, USA; ^6^William Beaumont Hospital, Division of Pulmonary and Critical Care, Department of Internal Medicine, Royal Oak, Michigan, USA

## Abstract

Adult intensivists have increasing exposure to individuals with congenital diseases surviving into adulthood. Solid knowledge bases and early recognition of the possible sequelae of congenital disorders are crucial in caring for these patients. We present a challenging case of shock and relapse of Diamond-Blackfan anemia in a 42-year-old man lost to follow-up for 18 years and highlighted the importance of healthcare transitions into adulthood and the challenges faced by health care systems to develop new strategies successfully transitioning complex pediatric patients to adult care.

## 1. Introduction

Diamond-Blackfan anemia (DBA) is a rare hereditary disorder secondary to a ribosomal protein gene mutation resulting in erythropoiesis failure, cancer predisposition, and associated congenital anomalies [1]. The incidence rate is 7 in 1,000,000 and is inherited mostly in an autosomal dominant fashion [1]. DBA presents within the first year of life with moderate to severe macrocytic anemia associated with mental retardation, hypertelorism, flat nasal bridge, high arched or cleft palate, triphalangeal thumb, and cardiac abnormalities [1–3].

The improvement in treatment modalities and diagnostics has improved the survival of patients with rare congenital diseases into adulthood by threefold between 2006 and 2016 [4–6]. Increased healthcare complexity highlighted the importance of transitions of care in decreasing hospital readmissions, system-wide financial burdens, and improving patients' outcomes [4]. In patients with rare diseases, these transitions of care are even more important as these patients' transition into adulthood [7].

Hereby, we report a case of high output cardiac failure and subsequent shock secondary to a relapse of DBA in a patient with a history of a repaired Tetralogy of Fallot lost to follow-up for 18 years highlighting the importance of close follow-up and transitions between pediatric and adult providers.

## 2. Case Presentation

A 42-year-old Caucasian male presented with worsening fatigue and dyspnea for 2 weeks. His medical history included a diagnosis of childhood Diamond-Blackfan anemia (DBA), for which he received corticosteroids until the age of 24 when he was lost to follow-up, and a Tetralogy of Fallot (TOF) with pulmonary atresia repaired at the age of 3 years with a right ventricle-pulmonary artery conduit. He denied any bleeding, and a complete review of systems was otherwise negative. His vital signs demonstrated a blood pressure of 138/90 mmHg, tachypnea (respiratory rate: 30 breaths/minute), tachycardia (pulse rate: 110 beats/minute), and a temperature of 36.4°C, and he was saturating 88% on a nonrebreather mask. He appeared in visible respiratory distress and pale and had evidence of a Klippel-Feil deformity, a triphalangeal thumb, and a harsh diastolic murmur over the left sternal border.

**Figure 1:**
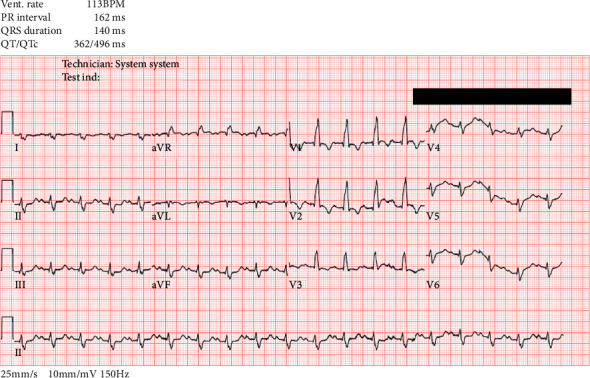
Initial electrocardiogram (EKG) demonstrating right bundle branch block, right axis deviation, and sinus tachycardia.

His labs demonstrated profound anemia with an hemoglobin of 2.5 g/dl, an elevated white blood cell count of 26.4 cells/mm^3^, and a normal platelet count. His serum creatinine was 1.02 mg/dl, and his serum lactic acid was 6.3 mmol/L. Liver chemistries demonstrated elevated aspartate aminotransferase of 1072 U/L, alanine aminotransferase of 1568 U/L, and total bilirubin of 3.2 mg/dl. His international normalized ratio (INR) was 3.2, and his activated partial thromboplastin time (aPTT) was 49.5 seconds. A brain natriuretic peptide (BNP) was elevated at 2942 pg/ml. A thyroid stimulating hormone (TSH) level was normal. A chest X-ray (CXR) demonstrated cardiomegaly and pulmonary edema. An electrocardiogram (EKG) demonstrated a right bundle branch block (RBBB), a posterior left fascicular block, and sinus tachycardia **(**Figure 1**)**.

**Figure 2:**
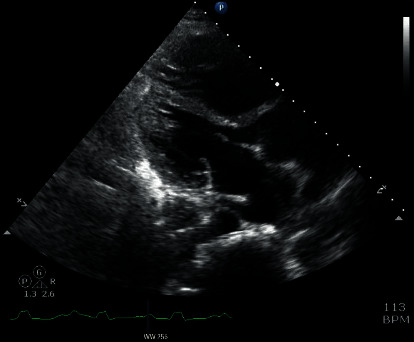
Transthoracic echocardiogram (TTE) demonstrating right sided ventricular hypertrophy.

**Figure 3:**
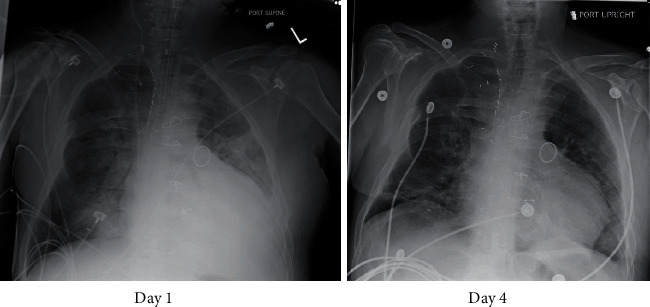
Initial plain chest film demonstrating acute pulmonary edema and repeat study at four days of hospitalization with improvement in volume overload. Congenital rib deformity and pulmonic valve ring are visible in both images.

**Figure 4:**
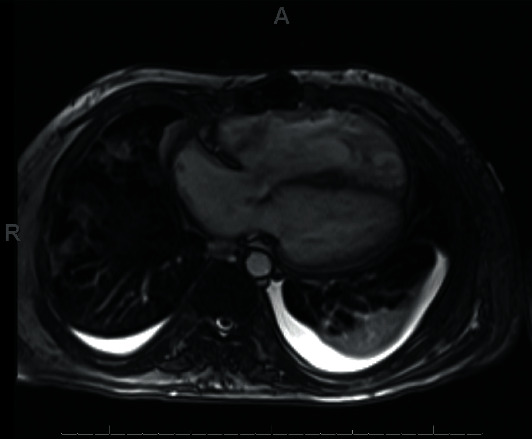
Cardiac magnetic resonance imaging demonstrating marked right ventricular enlargement.

Progressive worsening of clinical status with bilevel positive airway pressure (BiPAP) prompted intubation and mechanical ventilation. He was transfused 6 units of packed red blood cells (PRBC) with improvement of his hemoglobin to 8.8 g/dl. He received concomitant doses of furosemide to avoid further volume overload. He required norepinephrine for hemodynamic instability for 48 hours. A repeat echocardiogram demonstrated severe right ventricular enlargement **(**Figure 2**)**. He was extubated after three days of optimizing his volume and respiratory status, and his liver chemistries, INR, and serum lactate normalized.  Figure 3 compares an initial CXR and one performed at day 4. He was diuresed gently and discharged after 7 days of admission. A cardiac magnetic resonance image (MRI) showed a normal left ventricular ejection fraction (EF) of 55%, a severely enlarged right atrium and right ventricle (RV) with marked depression in right ventricular function (RV EF: 18%) (Figure 4**)**, and severe bioprosthetic pulmonary regurgitation. His anemia was attributed to a relapse of his DBA, and he was restarted on prednisone. On 2 years of follow-up, he continues to refuse a bone marrow transplant, is transfusion-dependent, and is planned for a percutaneous pulmonary valve replacement. On evaluation of reasons for loss to follow-up, the patient reported a traumatic experience receiving a bone marrow aspiration in his adolescence.

## 3. Discussion

DBA diagnosis requires a correlation between examination findings and laboratory abnormalities demonstrating macrocytic anemia, elevated hemoglobin F levels, high erythrocyte deaminase activity, and confirmation with bone marrow biopsy and gene karyotyping. DBA is associated with congenital heart disease (CHD) in 30% of patients with atrial and ventricular septal defects constituting the majority of associated anomalies [1–3]. Associated Tetralogy of Fallot (TOF) accounts for only 3.5% of associated anomalies, and pulmonary hypertension following TOF repair occurs in around 1% of patients and often seen in patients with major aortopulmonary collateral arteries [1–3]. DBA management requires cooperation and coordination between multiple specialties given the complexity of the disorder and its effect on different organs. High dose followed by maintenance steroids and supportive transfusions represent initial lines of therapy with bone marrow transplantation utilized as definitive therapy in patients failing to achieve or maintain remission [8, 9]. Pregnancy and viral infections can cause relapse of the anemia with the latter being a possible trigger in the reported patient [8, 9].

Our patient presented with severe anemia resulting in high-output cardiac failure precipitating shock in the setting of severe pulmonary hypertension following a repaired TOF. This case represents several missed opportunities for earlier intervention that could have prevented the significant morbidity that our patient endured because of loss to follow-up. Firstly, early recognition of DBA relapse and reintroduction of steroids could have treated his anemia. Additionally, serial monitoring for development of pulmonary regurgitation, pulmonary hypertension, and subsequent right ventricular failure prompting early consideration for pulmonic valve replacement.

Multiple factors contribute to fractured transitions of care into adulthood including improper planning and coordination and low adult provider comfort levels and familiarity with chronic and complex pediatric diseases [10]. Patient factors include psychological effects of chronic disease on children and adolescents and lack of access to advanced healthcare and resources in rural populations [7, 11]. Efforts to create safer transitions of care for pediatric patients transitioning into adulthood are needed to prevent additional morbidity and mortality.

## Additional Points

*Article Guarantor*. Zaid Imam, MBBS. *Presentation*. American Thoracic Society Conference 2018, San Diego, California, USA; poster presentation.

## References

[1] Vlachos A., Ball S., Dahl N. (2008). Diagnosing and treating Diamond Blackfan anaemia: results of an international clinical consensus conference. *British Journal of Haematology*.

[2] Vlachos A., Blanc L., Lipton J. M. (2014). Diamond Blackfan anemia: a model for the translational approach to understanding human disease. *Expert Review of Hematology*.

[3] Da Costa L., Narla A., Mohandas N. (2018). An update on the pathogenesis and diagnosis of diamond–blackfan anemia. *F1000Research*.

[4] American Academy of Pediatrics; American Academy of Family Physicians; American College of Physicians-American Society of Internal Medicine (2002). A consensus statement on health care transitions for young adults with special health care needs. *Pediatrics*.

[5] Mazzucato M., Visonà Dalla Pozza L., Minichiello C. (2018). The epidemiology of transition into adulthood of rare diseases patients: Results from a population-based registry. *International Journal of Environmental Research and Public Health*.

[6] Yasuhara J., Yamagishi H. (2015). Pulmonary arterial hypertension associated with tetralogy of Fallot. *International Heart Journal*.

[7] Touraine P., Polak M. (2018). Challenges of the transition from pediatric care to care of adults: “say goodbye, say hello”. *Endocrine Development*.

[8] Engidaye G., Melku M., Enawgaw B. (2019). Diamond blackfan anemia: genetics, pathogenesis, diagnosis and treatment. *Electronic Journal of the International Federation of Clinical Chemistry and Laboratory Medicine*.

[9] Vlachos A., Muir E. (2010). How I treat Diamond-Blackfan anemia. *Blood*.

[10] Okumura M. J., Heisler M., Davis M. M., Cabana M. D., Demonner S., Kerr E. A. (2008). Comfort of general internists and general pediatricians in providing care for young adults with chronic illnesses of childhood. *Journal of General Internal Medicine*.

[11] Li H., Ge S., Greene B., Dunbar-Jacob J. (2019). Depression in the context of chronic diseases in the United States and China. *Int J Nurs Sci.*.

